# Genetic Characterization of HPAI (H5N1) Viruses from Poultry and Wild Vultures, Burkina Faso

**DOI:** 10.3201/eid1304.061356

**Published:** 2007-04

**Authors:** Mariette F. Ducatez, Zekiba Tarnagda, Marc C. Tahita, Adama Sow, Sebastien de Landtsheer, Brandon Z. Londt, Ian H. Brown, Albert D.M.E. Osterhaus, Ron A.M. Fouchier, Jean-Bosco B. Ouedraogo, Claude P. Muller

**Affiliations:** * National Public Health Laboratory, Luxembourg; † Institut de Recherche en Sciences de la Santé, Bobo-Dioulasso, Burkina Faso; ‡ Veterinary Laboratories Agency, Surrey, England, United Kingdom; ‡ Laboratoire National de l’Elevage, Ouagadougou, Burkina Faso; § Veterinary Laboratories Agency, Surrey, England, United Kingdom; ¶ Erasmus Medical Center, Rotterdam, the Netherlands

**Keywords:** HPAI, H5N1, hooded vulture, poultry, Burkina Faso, dispatch

## Abstract

Genetic analysis of highly pathogenic avian influenza (H5N1) viruses from poultry and hooded vultures in Burkina Faso shows that these viruses belong to 1 of 3 sublineages initially found in Nigeria and later in other African countries. Hooded vultures could potentially be vectors or sentinels of influenza subtype H5N1, as are cats and swans elsewhere.

On February 7, 2006, the first African outbreak of highly pathogenic avian influenza (HPAI) (H5N1) virus was reported from a farm in Kaduna State, northern Nigeria. Since then, 7 other African countries, including Niger, Egypt, Cameroon, Burkina Faso, Côte d’Ivoire, Sudan, and Djibouti, have officially reported HPAI (H5N1) in poultry farms to the World Organization for Animal Health. On April 3, influenza A (subtype H5N1) was first confirmed in Burkina Faso. We genetically analyzed influenza A (H5N1) viruses from Burkina Faso poultry and the first gene sequences obtained from African wild birds, hooded vultures (*Necrosyrtes monachus*).

In March and April, cloacal and tracheal swabs from 3 adult hooded vultures were collected in Ouagadougou, Burkina Faso. The first of these birds of prey had dyspnea and neurologic signs; the second showed asthenia and locomotion problems. While only samples from these 3 vultures were available for sequencing, an additional 45 hooded vultures were found dead or sick throughout Ouagadougou from February to June 2006; symptoms in these birds included diarrhea, respiratory disorders, prostration, apathy asthenia, and ruffled feathers. Seventeen of these were confirmed influenza positive by rapid test (Influenza A&B test kit, BinaxNOW, Binax Inc., Portland, ME, USA). All birds of prey were collected as part of a passive surveillance program, and only birds sick enough to attract attention (and to be caught without special equipment) were sampled. In addition, swabs were collected from 1 domestic guinea fowl in Ouagadougou and from 4 adult backyard chickens from flocks with high numbers of deaths in Bobo-Dioulasso, Tenado, and Sokoroni, located ≤150 km from each other. The swabs of all birds were positive for HPAI (H5N1) virus, as evidenced by generic influenza A M-gene reverse transcription-PCR (*1*) and specific H5 PCR, as recommended by the European Union (http://eur-lex.europa.eu/LexUriServ/site/en/oj/2006/l_237/l_23720060831en00010027.pdf, 04/08/2006).

The hemagglutinin (HA) sequences of influenza A (H5N1) viruses from Burkina Faso clustered with recent western Asian, Russian, European, and African strains and are clearly distinct from southeastern Asian lineages (data not shown). Phylogenetic comparison of the HA1 genes from Burkina Faso with all African influenza A (H5N1) HA sequences available from GenBank showed that the Burkina Faso strains cluster together and with A/chicken/Ivory Coast/1787/2006 (Figure 1). Nucleotide differences between all 8 Burkina Faso HA1 sequences were relatively small (0.1% [1 nt] to 0.9% [10 nt]). HA1 sequences of viruses from 3 hooded vultures (A/hooded vulture/Burkina Faso/1–2-5346–10/2006) showed 0.2%–0.5% (3–6 nt) differences. A poultry virus from Côte d’Ivoire (A/chicken/Ivory Coast/1787/2006) was more similar to Burkina Faso strains than to other African strains, with a mean percentage nucleotide difference for HA1 of 0.5% (2–10 nt). HA1 sequences from the southwestern Nigerian farm (coded SO) (*2*) showed >1% nt differences when compared with the Burkina Faso strains. The maximum percentage nucleotide difference within African influenza A (H5N1) HA1 sequences ranged up to 1.8% (between A/chicken/Egypt/5611NAMRU3-AN/2006 and A/chicken/Sudan/1784/2006). Mean percentage nucleotide differences within Burkina Faso and within Nigeria (sequences from southwestern SO and BA farms and northern Nigeria combined) were similar, reaching 0.52% and 0.54%, respectively. Viruses detected in Niger, Sudan, and Egypt were more homogeneous, with ≤0.24% nucleotide diversity. In Nigeria, both the diversity and the phylogenetic pattern suggested at least 3 independently introduced influenza A (H5N1) lineages in the country (*2*). The branching of the Burkina Faso sequences (Figure 1) suggests that they emerged from a common ancestor, probably shared with other African avian viruses (A/chicken/Ivory Coast/1787/2006, A/chicken/Nigeria/641/2006, and strains from Sudan). Strains from Burkina Faso, northern Nigeria, Sudan, and Côte d’Ivoire constitute the putative African cluster C. The data presented in Figure 1 do not support the occurrence of multiple lineages of influenza A (H5N1) viruses in Burkina Faso. However, phylogenetic analysis further indicates that the African HPAI (H5N1) strains form 2 additional clusters. Cluster A contains the strains found in the southwestern Nigerian BA farm and includes all strains found in Niger. Cluster B includes the strains from the southwestern Nigerian SO farm and includes all Egyptian strains. The 3 sublineages correspond to those described previously in Nigeria (*2*) that now show a clear geographic distribution in Africa (Figure 2).

**Figure 1:**
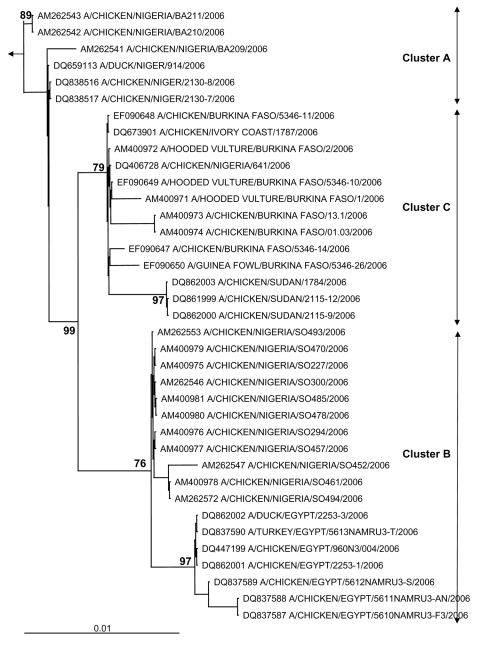
Phylogenetic tree for the hemagglutinin (HA) gene of African influenza A (H5N1) strains. The maximum likelihood method was used with 100 bootstraps and 3 jumbles (DNA-ML, Phylip version 3.6) to construct a tree for HA1 nucleotide sequences. Bootstrap values of major nodes are shown. The arrow points to the outgroup strain, A/goose/Guangdong/96. As detailed in the text, cluster C regroups highly pathogenic avian influenza (H5N1) strains from Burkina Faso, northern Nigeria, Sudan, and Côte d'Ivoire; cluster A regroups strains from a southwestern Nigerian farm (coded BA) and Niger; and cluster B regroups strains from a southwestern Nigerian farm (coded SO) and Egypt. The scale bar represents ≈1% of nucleotide changes between close relatives.

**Figure 2:**
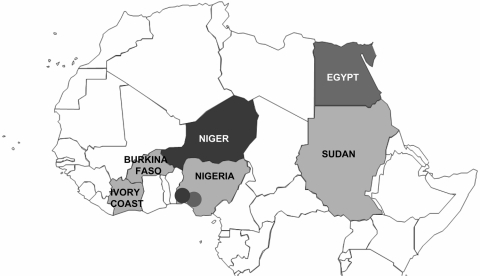
Northern equatorial Africa. Only countries where highly pathogenic avian influenza A (H5N1) sequences from avian species are available are named. Strain similarities are indicated as follows: light gray for countries with cluster I strains, black for countries with cluster II strains, and dark gray for countries with cluster III strains**.**

The amino acid sequence of the HA cleavage site of Burkina Faso strains, PQGERRRKKRG, was identical to that of the recent HPAI strains from western Asia, Russia, Europe, and Africa. The 4 strains had an ^222^Q-^224^G pattern (H5 numbering [*3*]), compatible with a preferential binding of the virus to α2,3 sialic acid (*4*), mostly found in avian strains. All other amino acid residues thought to be relevant for receptor binding (positions 91, 130–134, 149, 151, 179, 186, 190–191, and 220–225 [*3*]) were identical to those found in all western Asian, Russian, European, and African influenza A (H5N1) sequences as well as in HK/156/97. Therefore, we concluded that the Burkina Faso and other African strains do not contain mutations that facilitate binding to human α2,6 sialic acid receptors.

Although migratory birds in Egypt and a sparrowhawk (*Accipter nisus*) in Côte d’Ivoire were reported to be infected by H5N1 viruses (http://www.oie.int), this study is the first to describe the molecular characterization of HPAI (H5N1) viruses detected in African wild birds. We show that the H5N1 strains from northern Nigeria, Burkina Faso, Côte d’Ivoire, and Sudan probably evolved from a common ancestor. The vector of this transmission has not been identified. Virus transmission from domestic to wild birds has rarely been observed (*5**,**6*), but it appears to be a likely scenario since vultures feed on dead poultry and since during the same time (March 2006) an outbreak of HPAI (H5N1) occurred in an intensive farming system (http://www.oie.int/) in Ouagadougou, where infected vultures were also found. Moreover, the virus strains obtained from chickens and vultures in Ouagadougou are phylogenetically similar, as previously described. Hundreds of these birds can be observed around sites such as abattoirs or market places (*7*). Their feeding behavior could facilitate transmission within domestic poultry, and they could be involved in transmission from farm to farm, consistent with conventional mechanisms of spread through human activity. Moreover, these birds are in close contact with many other scavengers (e.g., hyenas, lions) with similar feeding habits. Hooded vultures also come in close proximity with humans since they are gregarious around human settlements and are used in traditional medicine, which adds to a small but potential risk for virus transmission to humans. Since these vultures often stay close to their local urban or semiurban feeding grounds, they may play a role primarily as short-range vectors, probably through mechanical transmission. In West Africa, some populations may also move north and south with the rains (*7*). Further studies are clearly required to better understand the potential role of vultures in the transmission of HPAI (H5N1) virus. Although vultures are clearly susceptible to this virus, their signs and symptoms could not be unequivocally attributed to subtype H5N1 alone. If these viruses cause severe disease in hooded vultures, these ubiquitous scavenging birds may simply be dead-end hosts. As they scavenge on many dead species, they may also function as conspicuous sentinels in Africa, similar to the role of raptors or swans in Europe or cats in Indonesia (*8*).

Proper disposal of infected carcasses must be carefully enforced on affected farms to avoid primary infections of carrion feeders. The role of hooded vultures and other scavenger birds as vectors of HPAI (H5N1) to other wild birds, poultry, and mammals, including humans, as well as their potential role as sentinels, requires further investigation in Africa.
